# Microanatomical study of the human pancreatic made on necroptic pieces

**Published:** 2012

**Authors:** GI Purice, G Onose

**Affiliations:** * “Carol Davila” University of Medicine and Pharmacy, “Bagdasar Arseni” Clinical Hospital, Rehabilitation Department, Bucharest, Romania; ** “Bagdasar – Arseni” Clinical Hospital, Rehabilitation Department, Bucharest, Romania

**Keywords:** pancreas, micro anatomic study, micro vascularization

## Abstract

The pancreas is a bulky gland, with mixed secretion, exocrine and endocrine, attached to the duodenum, participating through its secretions in carbohydrates digestion and metabolism. For a long time, it was considered a mysterious organ, with an inaccessible, examining and exploring due to its deep retroperitoneal position.

We intend to make a comparative analysis of pancreatic microanatomy, between the examination of the necroptic-collected pieces and the ex vivo pieces, intraoperatively, with the patient’s prior consent. We aimed to deepen the qualitative micro anatomic study on the pancreas parts of dissection, and quantitative study of the vessels micro anatomic normal pancreatic body.

The methods and techniques used were the anatomical study through dissection and intraoperative and qualitative micro anatomic study by making blades of pathological sample products taken from patients: extemporaneous and microscope examination.

## Introduction

**Fig. 1:**
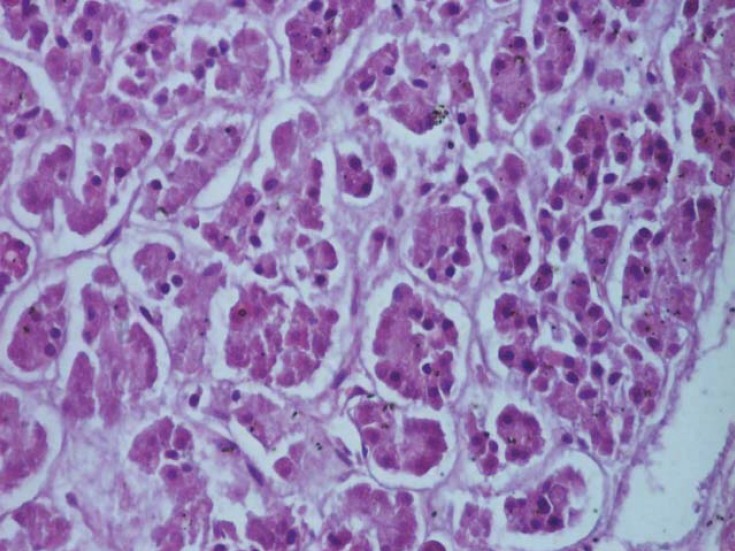
Fragment of the exocrine pancreas, from the tail, which shows acini of variable size and shape (H & E, ob. 20x).

The pancreas is a mixed gland (amphicrania) with double secretion, having two components [**1-3**]: an endocrine component, consisting of epithelial tissue endocrine placed as islands, islets of Langerhans, called insular pancreas or endocrine pancreas; and an exocrine component, consisting of exocrine epithelial tissue, arranged as acini, called the exocrine pancreas. The exocrine pancreas occupies most of the gland [**4,5**] (**Fig. 1**).

The pancreas shows a thin conjunctive capsule, with a weak structure, represented by a dense semiordered tissue. Stroma, within the pancreas, having a weak structure as well, has a loose connective tissue that supports the parenchyma elements building up the lobules. The stroma presents vascular-connective tissue septa, originating from the capsule [**5**].

**Fig. 2:**
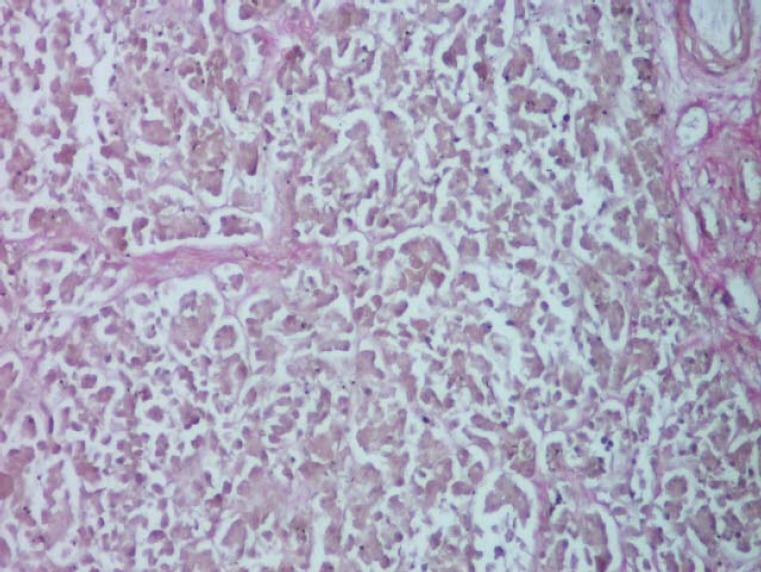
Disposition of interlobular septa containing vascular elements of different dimensions (color van Gieson's method, ob. 10x).

**Fig. 3:**
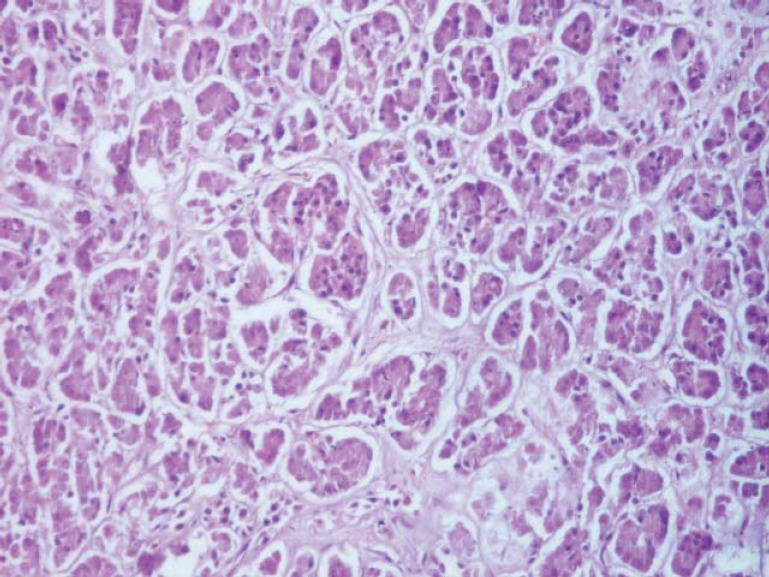
Exocrine pancreatic tissue, highlighting the secretory component (serous acinii) (H & E, ob. 10x).

The exocrine pancreas (pars exocrina pancreatis) is mostly present at the level of the gland (97-99%) [**5**]. The glandular organ, of a tubulo-acini type, serozymogenic [**5**], shows the ancreatic lobes, separated by connective tissue, and each lobes have pancreatic lobules (lobulus pancreaticus) separated by interlobular septa (interlobular septum) (**Fig. 2**). The lobule has a secretory component (serous acinii) (**Fig. 3**) and an excretory one (ducts) [**5,6**].

The pancreatic acini (acinus pancreaticus) are separated from each other by a loose connective tissue with blood vessels, lymph, nerves and ducts of excretion, tissue that is continued by that of the interlobular septa.

The acini have excretory ducts and acinar secretory pyramid cells containing zymogen granules that are placed on a basement membrane, reticular, in the periphery. Under the basal membrane of the acini, the mioepithelial cells are branched. In electron microscopy, the basal membrane is quite rich in rough endoplasmic reticulum (ergastoplasm) since this area contains a large number of mitochondria. Secretion granules (zymogen) pass from the rough endoplasmic reticulum, into the Golgi complex, where they will be converted to small vesicles, which shall be subsequently excreted.

The canalicular system of pancreatic lobules present: centro-acini ducts, which leave from the acini and consist of flattened cells; intercalary ducts, composed of cuboid cells and intralobular and interlobular ducts consisting of tall epithelium.

The interlobular ducts are joined together, then open into the channels of a higher caliber in Wirsung's pancreatic duct (ductus pancreaticus), as well as in the Santorini's accessory pancreatic duct (ductus pancreaticus accesorius) [**4-6**].

**Fig. 4:**
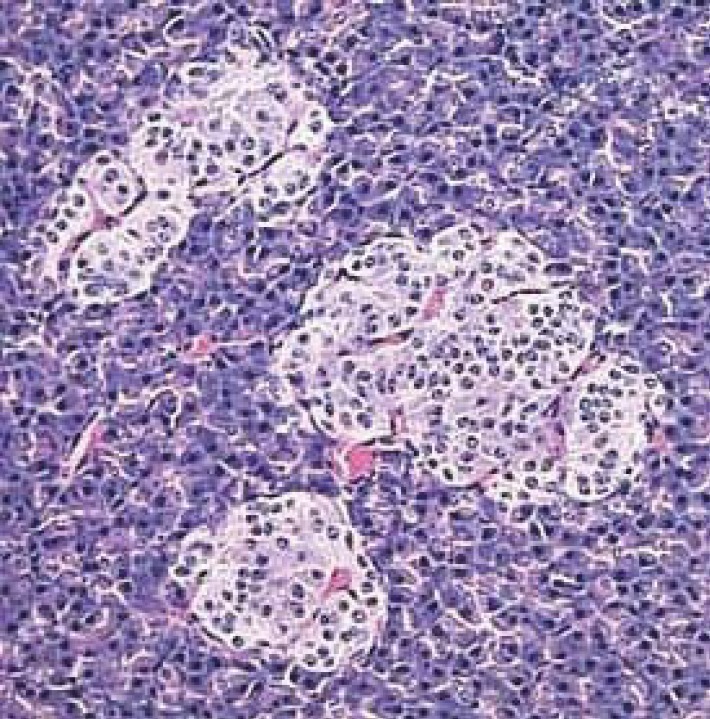
Fragment of the endocrine pancreas, which has three islands of Langerhans cells, by nr. [**7**].

The endocrine pancreas (pars endocrina pancreatis) (1-3%) or insular pancreas is composed of islands of pancreatic cells (insula pancreatica), called the islets of Langerhans scattered among glandular acini, being reduced in number at the level of the head and body of pancreas and more numerous in the tail of the pancreas (**Fig. 4**) [**5,6**].

The islets of Langerhans are separated by a reticulo-capillary network. Prismatic cell cords are anastomose to one another, being composed of α cells and β cells. The islets are surrounded by a very fine network of connective tissue, do not contain ducts and have several capillaries, called fenestrated capillaries. In terms of cytological aspects: alpha cells / A (20%) are to be found on the periphery of the islets and secret glucagon [**5**]; beta cells / B (70%) are widespread in the entire insular mass, and in a larger number within the center of islets of Langerhans [**6**], secrete insulin; delta cells / D (5%) are dispersed in the entire insular mass, and are secreting cells of gastrin, somatostatin, which prevent the release of insulin and glucagon and decrease smooth muscle contraction in the gallbladder and intestine [**5,6**] and the cells C (1%) are clear, agranular cells [**4-6**].

## Materials and methods

**Fig. 5:**
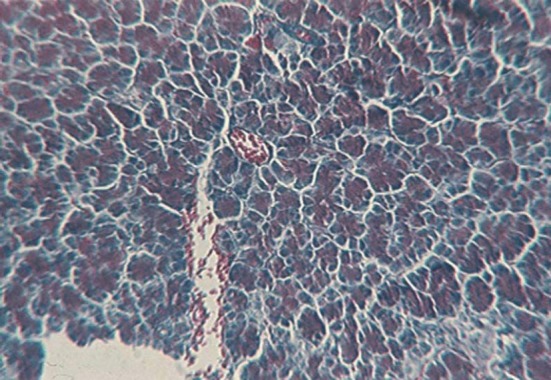
Section through the body of the pancreas in order to evidentiate the vascular device (Novelli stain, ob. 10x).

The micro anatomical study was performed on pancreatic tissue fragments collected from the main body segments during the first 24 hours of death. Legislation and methodology did not allow carrying out necroptic tests on more freshly collected samples. For this reason, the micro-anatomical study will be completed later, in another phase of the research, with pancreatic tissue harvested intraoperatively. The tissue fragments were processed by paraffin technique, stained by usual and special methods, the slides being qualitatively examined afterwards for the overall look and in order to detect any pathological lesions. Among the specific methods, quite useful is the Novelli technique, using Ewans blue which highlights the vascular device that can be easily computer quantified during the next phase of the study (**Fig. 5**).

**Fig. 6:**
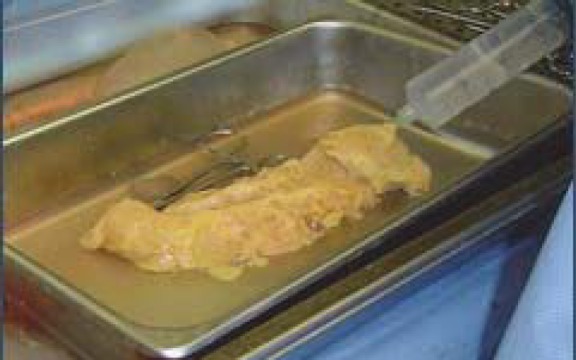
Methodology of pancreatic vessels injection

**Fig. 7:**
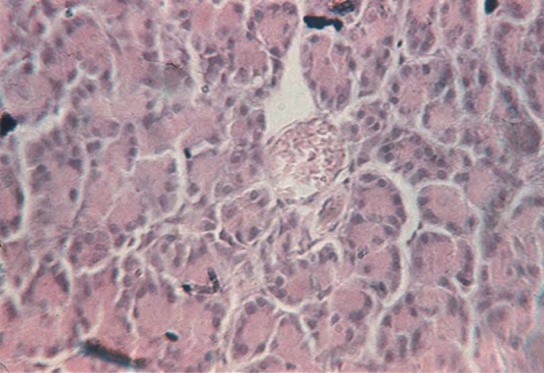
Section through the pancreas after injection of Chinese ink (H & E, ob. 20x).

In five cases, the organ has been specifically processed for the study of micro-vascularization: the fresh sample has been injected within the main arteries using a solution of Chinese ink and gelatin (**Fig. 6**); then, the tissue fragments have been processed by paraffin technique and H & E stained; the dye entered the vascular system, occupied all lumina and became obvious on all the sections (**Fig. 7**).

## Results and Discussions

1. Micro-anatomical aspects of quality.

The microscopic examination of fragments taken from different segments of the pancreas did not reveal various qualitative aspects of pancreatic tissue.

Thus, in all cases, the conjunctive capsule is thin, consisting of dense connective semi-ordered tissue.

**Fig. 8:**
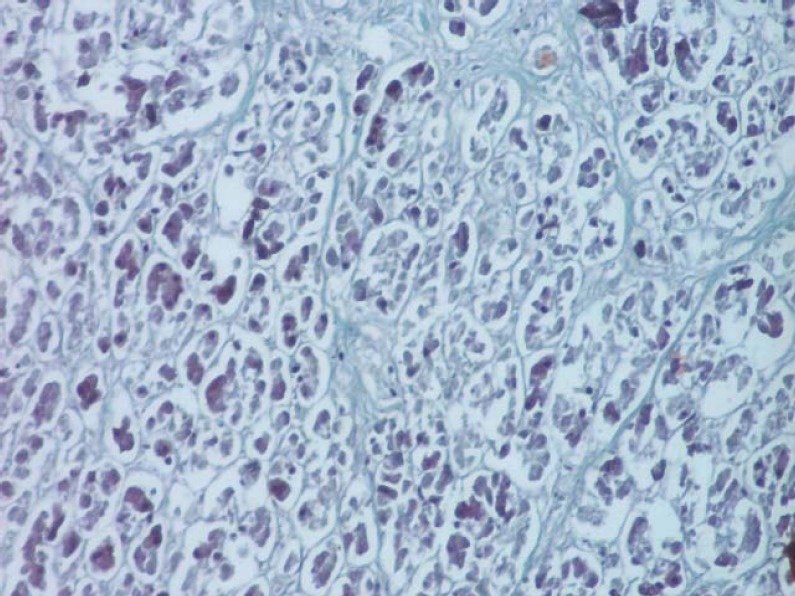
Section through the body of the pancreas harvested from a person of 63 years, with abundant stromal tissue, which separates acinii ( Szekely stain, ob. 10x).

In all its parts, the stroma of the organ is composed of loose connective tissue that separates the parenchyma elements of the lobules. There are numerous conjunctive vascular nervous septa originating from the capsule, which are distributed in all organ parts. In cases of elderly people, over 60 years old, the stroma is denser, the connective tissue more abundant, further studies having to stereologically determine the percentage they occupy (**Fig. 8**).

**Fig. 9:**
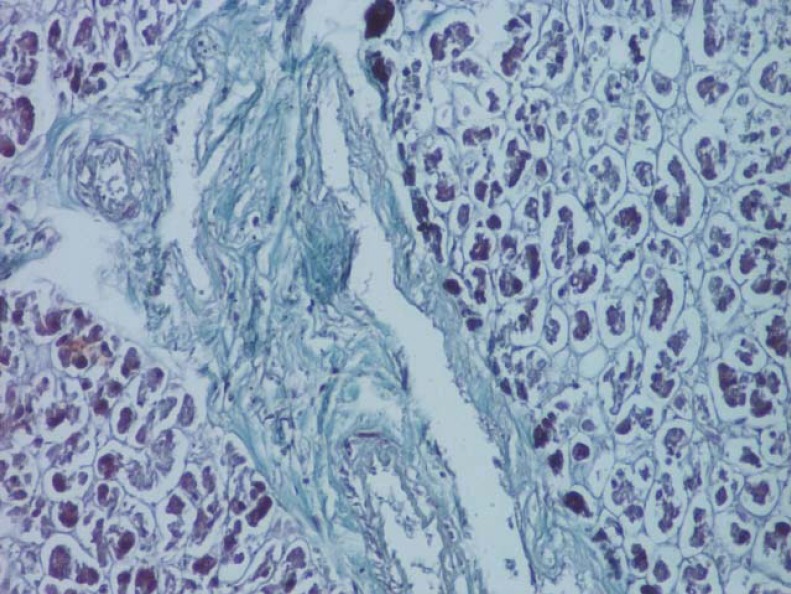
Section through the head of the pancreas affecting one interlobular septa (Szekely stain, ob. 20x).

**Fig. 10:**
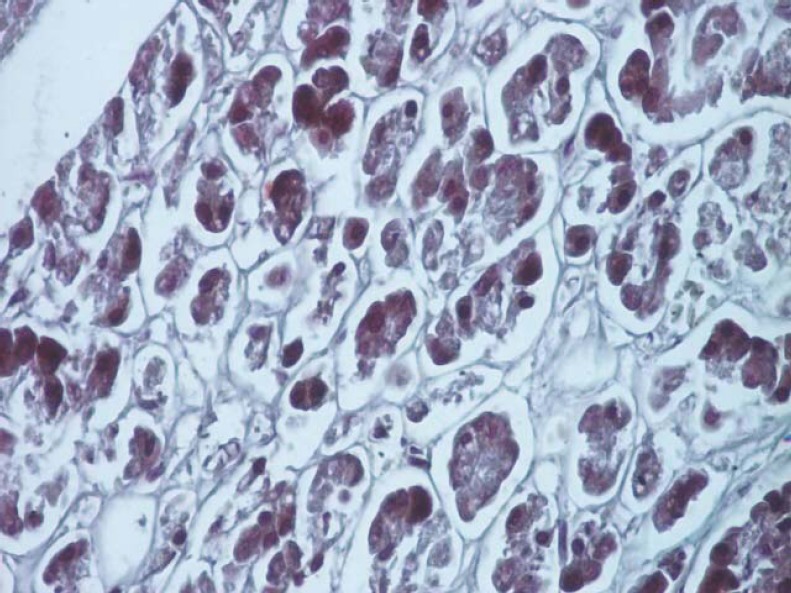
Stromal tissue with a periacinar disposition at the level of the head of the pancreas (Szekely stain, ob. 40x).

The exocrine parenchyma is composed of pancreatic acini separated from each other by a loose connective tissue, through which blood vessels, lymph, nerves and excretion ducts pass, continued by the interlobular septal tissue (**Fig. 9**) and by the periacinar tissue (**Fig. 10**).

The pancreatic acini are formed by the pyramidal secretory acinar cells (**Fig. 3**), containing zymogen granules that are placed on a reticular basement membrane, very fine, peripheral, which, together with the opposite side, converge to the central lumen of acini. The aspect of the lumen varies depending on the functional state of the organ, being reduced during the resting period and relaxed during the activity period. The microscopic examination of the ductal system of pancreatic lobules shows a different structure depending on the duct type: the central acinar ducts are lined with flat cells, cuboid cell intercalary ducts, while intralobular and interlobular ducts have a high or columnar epithelium.

2. The micro-irrigation of the pancreas

**Fig. 11:**
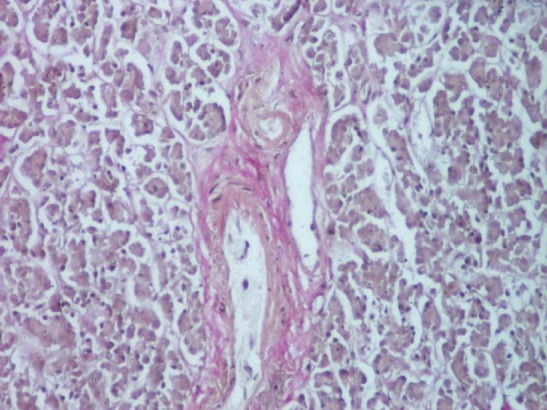
Arterioles present in the interlobular septa (van Gieson's stain method, ob. 20x).

**Fig. 12:**
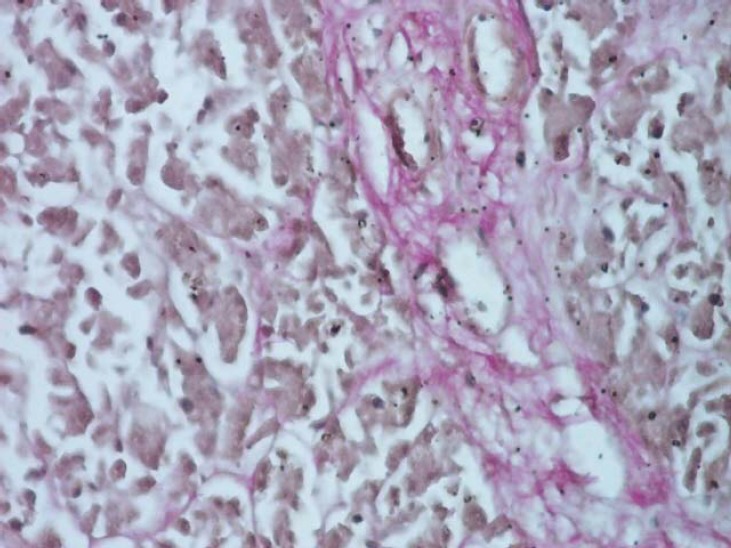
Interlobular septa with numerous vessels in the body of the pancreas (van Gieson's stain method, ob. 20x).

**Fig. 13:**
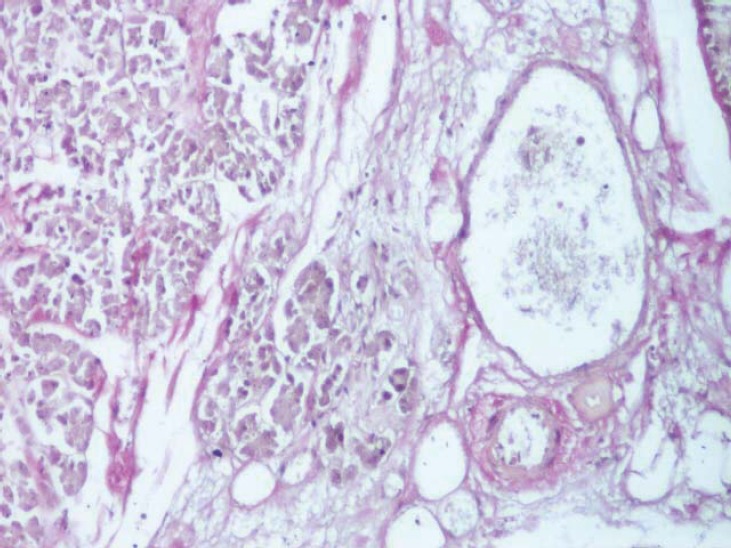
Dimensional variability of subcapsular vessels (van Gieson's stain method, ob. 10x).

**Fig. 14:**
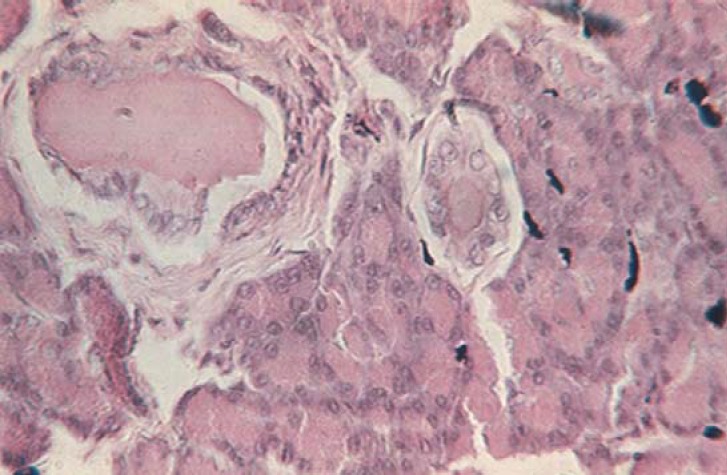
Section made through the pancreas after injection of China ink, highlighting several vessels (H & E staining, ob. 40x).

Within the pancreas, arteries branch off successively at the level of the septa into interlobular branches, out of which further intralobular branches get loose. These branches pass through the connective tissue between the acini, creating arterioles and a rich capillary periacinar network (**Fig. 11**, **Fig. 12**, **Fig. 13**). Since all the vessels go along the connective tissue branches, and vice versa, we may consider that portvas connective structures organize, playing an essential role in this organ with dual function that requires a particular topography of the vascular device (**Fig. 14**).

**Fig. 15:**
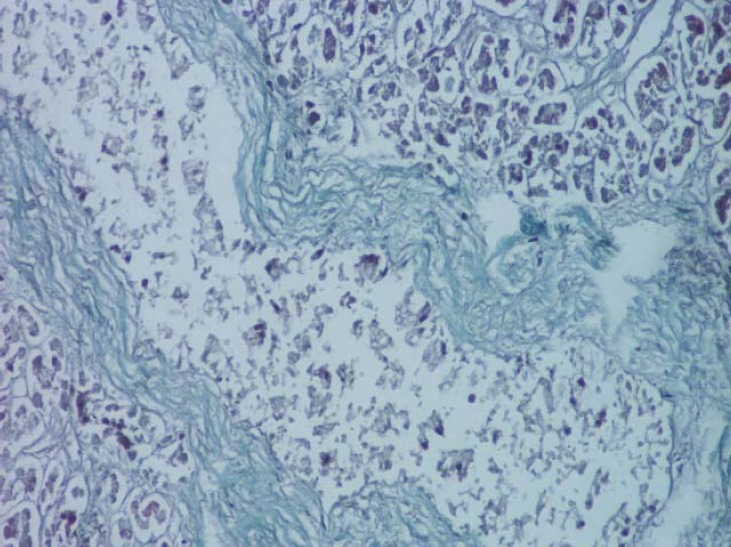
Section through the body of the pancreas, affecting obliquely an arteriola (Szekely stain, 10x).

Within the pancreas, arteries create arteriolar branches (**Fig. 15**) for the capillary network in the wall of excretory ducts. Arterial branches of the pancreas build up multiple arteriovenous anastomoses and arteriovenous anastomoses. Pancreatic islets are highly vascularized, crossed by large capillaries. Each islet has one or two arterioles, which may surround the island.

Arterioles, in turn, create a special network of capillaries, dilated, dispersed among the cellular cords, showing vascular glomeruli. Insular capillaries have a larger size larger as compared with the rest of the gland. Through them, the pancreatic hormones such as glucagon and insulin, go directly into the blood, which gives the islets their endocrine character.

The micro-anatomical structure of the pancreas favours the dissemination of a tumor due to fibrocollagenous system well represented throughout the body, making up the *portvas *and *portduct *structures that create pathways and directs the movement and progression of tumor cells.
